# Investigation of Localized Skin Temperature Distribution Across the Transtibial Residual Limb

**DOI:** 10.33137/cpoj.v4i1.35070

**Published:** 2021-01-12

**Authors:** K. Ghoseiri, M. Allami, J.R. Murphy, P. Page, D.C. Button

**Affiliations:** 1 School of Human Kinetics and Recreation, Memorial University of Newfoundland, St. John's, Newfoundland, Canada.; 2 Department of Orthotics and Prosthetics, School of Rehabilitation Sciences, Hamadan University of Medical Sciences, Hamadan, Iran.; 3 Janbazan Medical and Engineering Research Center (JMERC), Tehran, Iran.; 4 Faculty of Medicine, Memorial University of Newfoundland, St. John's, Newfoundland, Canada.; 5 Department of Physical Therapy, Franciscan University, Baton Rouge Louisiana, USA.

**Keywords:** Amputees, Skin Temperature, Thermography, Amputation Stumps, Artificial Limbs, Prosthesis Design, Residual Limb

## Abstract

**BACKGROUND:**

Interventions to resolve thermal discomfort as a common complaint in amputees are usually chosen based on the residual limb skin temperature while wearing prosthesis; whereas, less attention has been paid to residual limb skin temperature while outside of the prosthesis. The objective of this study was to explore the localized and regional skin temperature over the transtibial residual limb (TRL) while outside of the prosthesis.

**METHODOLOGY:**

Eight unilateral transtibial adults with traumatic amputation were enrolled in this cross-sectional study. Participants sat to remove their prostheses and rested for 30 minutes. Twelve sites were marked circumferentially in four columns (anterolateral, anteromedial, posteromedial, and posterolateral) and longitudinally in three rows (proximal, middle, and distal) over the residual limb and used for attachment of analog thermistors. Skin temperature was recorded and compared for 11 minutes. Furthermore, the relationship of skin temperature with participants’ demographic and clinical characteristics was explored.

**FINDINGS:**

The whole temperature of the TRL was 27.73 (SD=0.83)°C. There was a significant difference in skin temperature between anterior and posterior columns. Likewise, the distal row was significantly different from the proximal and middle rows. The mean temperature at the middle and distal zones of the anteromedial column had the highest and lowest skin temperatures (29.8 and 26.3°C, p<0.05), respectively. The mean temperature of the whole TRL had no significant relationships (p>0.05) with participants’ demographic and clinical characteristics.

**CONCLUSIONS:**

An unequal distribution of temperature over the TRL was found with significantly higher and lower temperatures at its anterior column and distal row, respectively. This temperature pattern should be considered for thermoregulation strategies. Further investigation of the residual limb temperature with and without prosthesis, while considering muscles thickness and blood perfusion rate is warranted.

## INTRODUCTION

Critical factors in the successful use of a prosthesis include skin integrity of the residual limb, skin health, and skin hygiene.^1,2^ Skin irritation, ulceration, dermatitis, and excessive sweating are common complaints of amputees who use prostheses for their daily activities.^3,4^ Heat and moisture that become trapped inside the socket lead to a jeopardizing, unpleasant, and infectious environment for amputees, which dramatically decreases the quality of life, satisfaction and use of the prosthesis, and social participation.^3,5,6^ In dysvascular and neuropathic patients, any area of the skin with 2°C or more increased temperature than adjacent areas has an increased risk of ulceration^7^; therefore, localized skin temperature is an indicator of a potential skin breakdown.

The transtibial residual limb (TRL) skin temperature measurements with the prosthesis demonstrated unequal heat buildup over different anatomical locations.^8,9^ Various scenarios are conceivable for unequal heat buildup over the TRL. A scenario could be referred to as the heterogeneous structure of the TRL, consisting of different underlying tissues with different thicknesses, blood perfusion rates, metabolic activities, and thermal characteristics.^9^ In another scenario, it could be referred to as socket and liner insulating nature, their materials characteristics, and their fit issues that may lead to higher frictions at the interface with the skin.^6,10^ Therefore, thermal discomfort with prostheses is not solely related to the socket and liner.

Previous investigations were mostly focused on controlling the temperature while prostheses were donned. Thus, most techniques for dealing with heat buildup addressed heat issues for inside prostheses. Recently, some developments in prosthetic components have been done to address thermal discomfort inside prostheses. For instance, the Silcare Breathe Cushion (Blatchford, UK) and Soft Skin Air (Uniprox, Germany) are perforated liners that permit air and moisture transfer from skin to the outer surface of the liner.^11,12^ Likewise, the SmartTemp liner (The Ohio Willow Wood, USA) has phase-change material inside its silicon structure, which permits energy storage and release in response to increased and decreased temperature, respectively. Temperature storage happens by changing the physical state from solid to liquid, whereas temperature release happens reversely.^13^ Thermoregulatory systems are smart components that could be mounted on prosthetic sockets. Some thermoregulatory systems were introduced in prior research with promising outcomes that could be commercialized once their electric power and weight issues are being resolved.^14,15^ However, to resolve thermal discomfort in people with TRL through prosthetic development, a comprehensive knowledge of temperature distribution over the residual limb is required.^11,14–16^ Understanding the baseline temperature distribution over the TRL without any external intervention such as socket or liner, may facilitate prosthetic design and technological development around natural residual limb temperature.

Skin temperature of TRL could be investigated using temperature sensors, thermography cameras, and virtual methods (i.e., mathematical modeling of the residual limb) during rest and activity.^5,8,9,17–19^ Temperature recording using thermistors is a complicated process inside the prosthesis. It needs control of the ambient temperature and mitigation of potential intervening parameters like inconsistent socket and liner characteristics, internal/external pressure on sensors, sensor wire breakdowns, movement artifacts, and decalibration. Therefore, measuring TRL skin temperature without prosthesis donned may provide more accurate, reliable results. Interestingly, few studies have measured TRL skin temperature without a prosthesis despite the more extensive literature about heat buildup inside the prosthesis.^19–21^ Perhaps such studies could provide a better understanding and insight into thermal discomfort in people with amputation. Although rarely investigated in people with amputation, some temperature control techniques and exercises for able-bodied people^22^ could be used in people with transtibial amputation pending baseline temperature distribution over the residual limb.

Furthermore, different study designs and unique characteristics of the amputee population make it difficult to compare the results between studies; therefore, further investigation of the TRL thermal pattern is needed. The present study aimed to establish a baseline of the TRL temperature distribution while the prosthesis was removed. In addition, relationships among demographic and clinical characteristics with residual limb temperature were explored.

## METHODOLOGY

### Participants

Eight male veterans were enrolled in this study based on a purposive sampling method. A list of all potential veterans with transtibial amputation living in the Hamadan province of Iran was excerpted and provided by the Veterans and Martyrs Affair Foundation (VMAF) from their comprehensive national database of about 500,000 Iranian veterans and martyrs.^23^ All veterans who met the study inclusion criteria were invited by a phone call to participate in this study. The inclusion criteria were: (**1**) unilateral TRL with at least 25 cm length from knee axis, (**2**) traumatic amputation, (**3**) age between 18-60 years, (**4**) at least two years of experience of prosthesis use, (**5**) existence of intact skin of the residual limb without any ulceration based on medical examination. The exclusion criteria were (**1**) existence of any medical comorbidities that may alter sensation/ thermoregulation (e.g., neurological, cardiovascular, and endocrine), (**2**) smoking for at least 30 minutes before starting the experiment,^24^ (**3**) alcohol drinking and medication use on experiment day, (**4**) impaired thermal sense in the residual limb based on clinical examination,^25^ (**5**) use of antiperspirant sprays, powder, and lotions on the skin of the residual limb on experiment day. After a full description of the study aims and procedures, written informed consent was obtained from participants before enrollment. All aspects of the study were approved by the ethics committee of the Hamadan University of Medical Sciences (IR.UMSHA.REC.1394.333).

### Temperature measurement over the transtibial residual limb

**Figure 1:**
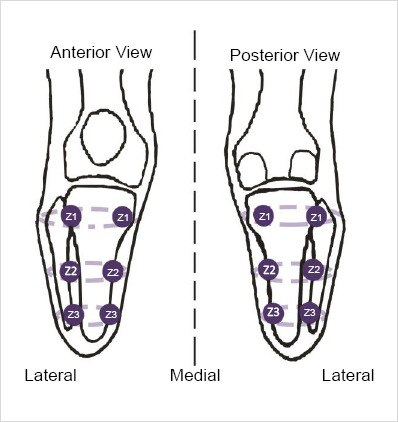
Temperature measurement sites over the transtibial residual limb

Twelve sites were marked circumferentially in four columns (anteromedial, anterolateral, posteromedial, and posterolateral) and longitudinally in three rows (proximal, middle, and distal) over the residual limb to provide attachment sites of thermistors.^8^ Attachment sites were longitudinally labeled Z1 to Z3 from proximal to distal and were marked at a constant distance to each other (Figure 1). In the longitudinal view, the distance from the knee center to the distal end of the residual limb was measured for each patient, divided by four to determine the appropriate distance between sensors. In the circumferential view, two columns of sensors were considered in the anterior and two in the posterior part of the residual limb. Columns were located on major muscle masses in line with prior similar studies.^5,17^ A portable thermoregulatory system designed, fabricated, and tested in a previous study was used for data collection.^20^ Twelve analog NTC (negative temperature coefficient - NXFT15XH103, Murata Manufacturing Co. Ltd., Japan) thermistors were calibrated and then attached to the skin using adhesive tape.^20^ Each thermistor was wired to a small amplifier board and connected to the input port of an Arduino Duemilanove (Arduino, Italy) microcontroller board. A seven volts lithium-ion battery with a nominal capacity of 2.2 Ah was used to supply the necessary power for thermistors and the microcontroller.

### Experimental setup

All data collection was done on three consecutive days, from 8 a.m. to 1 p.m. Participants sat to remove their prosthesis and rested for 30 minutes to become familiar with the laboratory environment and adapt to the ambient temperature. Demographic and clinical characteristics of participants were collected, and inclusion/exclusion criteria were verified. Thermistors were then attached to the marked sites over the residual limb. During one session, the localized skin temperature of the residual limb was recorded for 11 minutes at the ambient temperature of 23°C.

### Data and statistical analysis

Statistical analyses were computed using IBM SPSS software (Version 22.0, IBM Corp, New York, NY). The normality distribution of temperature data was assessed and determined by the Shapiro-Wilk test. The mean temperature of the residual limb was calculated at each zone compared to the mean temperature at other anatomical zones. Also, the overall temperature of the residual limb was determined. The grand mean or pooled mean, which was the mean of all residual limbs’ average temperature, was calculated. The mean temperature at each zone was compared to the residual limb’s mean temperature and the grand mean temperature using one-sample t-tests. Levene’s test for homogeneity of variances was explored between columns and rows over the TRL.

Since there were equal variances, parametric one-way analysis of variance (ANOVA) was used to examine the variability of temperature in columns and rows. Tukey post hoc analysis was used to identify differences among columns, as well as circumferential rows. Pearson's correlation coefficient and partial eta squared were calculated to explore the potential relationship of the residual limb’s average temperature with quantitative and nominal characteristics of participants. Significance for all data was defined as p<0.05, and all data are reported as mean ± SD (standard deviation).

## RESULTS

### Demographic and clinical characteristics of participants

Twenty-eight veterans volunteered to participate and attended a pre-screening of their adaptability with the inclusion/exclusion criteria. Twenty veterans were excluded from the study because of the existence of skin irritation of the residual limb (n=3), uptaking medications (n=6), associated medical comorbidities (n=9), and applying lotion over the skin of the residual limb (n=2). Therefore, all tests were done with eight veterans. The demographic and clinical characteristics of participants are presented in Table 1. Participants had a mean age of 40.3 (SD=8.4) years. For employment status, fifty percent of participants were employed and had a job; the remaining participants were retired or unemployed and received compensation and pension from VMAF based on their disability rating. The average time after amputation and experience of using a prosthesis were 19.3 (SD=9.6) and 18.9 (SD=9.8) years, respectively. Exoskeletal prosthesis use was the same as endoskeletal prosthesis among participants; however, polyfoam liner was more popular than silicon/gel liners. Average daily prosthesis use was 10 (SD=3.5) hours.

**Table 1:** The demographic characteristics of participant veterans (N=8)

Variable	Data Range	Mean	Standard Deviation (SD)
Age	23-51	40.3	8.4
Weight (kg)	60-92	75.8	8.8
Height (cm)	165-178	170.6	4.3
BMI (kgm^-2^)	19.6-31.8	26.1	3.5
Time after Amputation (y)	4-30	19.3	9.6
Years of Prosthesis Use (y)	3-30	18.9	9.8
Daily Prosthesis Use (h)	7.5-18	10	3.5
Employment Status	E: n=4 ^*^Un-E: n=4		
Amputation Side	R: n=5 L: n=3		
Type of Prosthesis	Ex-P: n=4 En-P: n=3 En-S: n=1		

E: Employed; *Un-E: Unemployed (Retired or unemployed veterans and veterans who received compensation and pension from Veterans and Martyrs Affair Foundation (VMAF) based on their disability rating considered unemployed); R: Right side; L: Left side; Ex-P: Exoskeletal with polyfoam liner; En-P: Endoskeletal with polyfoam liner; En-S: Endoskeletal with silicone/gel liner.

### Temperature measurement over the transtibial residual limb

**Figure 2:**
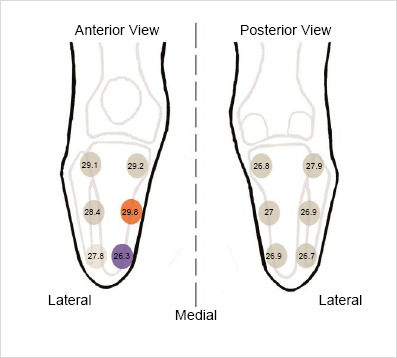
Average of the recorded temperature (°C) at each anatomical zone over the TRL, and the highest (red) and lowest (blue) temperature zones.

Figure 2 shows the localized mean temperature at different anatomical zones. The highest temperature was recorded at the middle portion of the anteromedial region of the TRL. The lowest temperature was recorded at the distal end of the anteromedial part of TRL (Figure 2).

The mean, SD, and SE (standard error) of the skin temperature recorded by the thermistors for each zone are presented in Table 2. The mean temperature of the residual limb and the grand mean temperature of all residual limbs were calculated and shown in Table 2. The grand mean of the skin temperature for all residual limbs was 27.7°C.

**Table 2:** The recorded temperature by the twelve thermistors, their mean and SD per zone and participant, and temperature comparison between the grand mean of all residual limbs and mean temperature at each zone.

Case ID	Regional residual limb temperature												Whole residual limb temperature				
	Anterolateral			Anteromedial			Posteromedial			Posterolateral							
	Z1	Z2	Z3	Z1	Z2	Z3	Z1	Z2	Z3	Z1	Z2	Z3	*Mean*	*SD*	*SE*	*t^1^*	*P*
1	27.8	27.7	26.4	28.4	28.8	24.8	27.4	27.6	24.8	27.1	26.1	24.8	26.8	1.4	0.4	0	1
2	28	27.9	26.6	27.7	29.6	26.5	29.2	26.5	24.8	27.5	24.4	24.3	26.9	1.7	0.5	0	1
3	30.6	26.6	28.5	31.1	29.6	28	24.7	25.7	28	27.7	26.8	27.1	27.9	1.9	0.5	-0.1	1
4	29.2	29.3	26.7	28.2	29	25.5	27	24.4	26.5	28.4	25.8	25.2	27.1	1.7	0.5	0	1
5	30	29.3	27.9	30	29.8	25.9	24.1	27.8	26.4	29.8	28.5	27.7	28.1	1.9	0.5	0	1
6	29.7	28.8	28.7	28.8	29.8	26.8	29	26.8	27.1	28.6	27.6	27.3	28.2	1.1	0.3	0.2	0.9
7	27.8	29.4	27.2	28.4	30.2	25.8	26.5	27.2	26.3	26.3	26.8	26.9	27.4	1.3	0.4	0	1
8	29.7	28.5	30.5	31.1	31.9	27.1	26.7	29.4	31.4	28.2	28.9	30.4	29.5	1.7	0.5	0	1
*Mean*	29.1	28.4	27.8	29.2	29.8	26.3	26.8	27	26.9	27.9	26.9	26.7	27.7	0.8			
*SD*	0.1	0.9	1.3	1.3	0.9	0.9	1.7	1.4	2	1	1.4	1.8					
*SE*	0.4	0.3	0.5	0.5	0.3	0.4	0.6	0.5	0.7	0.4	0.5	0.7					
*t^2^*	3.6	2.1	0.2	3.2	6.4	-4	-1.4	-1.4	-1.1	0.6	-1.6	-1.4					
*P*	0.01*	0.08	0.85	0.02*	0.00*	0.01*	0.21	0.20	0.33	0.54	0.15	0.20					

Z: zone; SD: standard deviation; SE: standard error; t^1^: one sample t statistics in comparison to mean temperature of the residual limb; t^2^: one sample t statistics in comparison to the grand mean temperature of all residual limbs; P: P-value; *: the difference is statistically significant (*p<0.05*).

**Figure 3:**
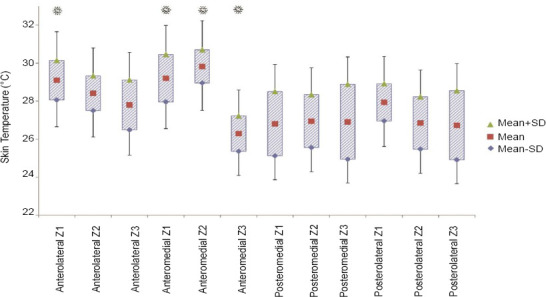
The mean value of the recorded temperature at each zone over the residual limb (*: the difference is statistically significant (p<0.05) based on one sample t-test in comparison of the grand mean of all residual limbs’ temperature and the mean temperature at each zone).

There was no significant difference between the mean temperature at each zone and the whole residual limb's temperature. Whereas, the comparison between the grand mean temperature of all residual limbs and the mean temperature at each zone indicated a significant difference in four zones (Figure 3).

The variability of the mean temperature was significant among four columns (F(3,92)=6.09, p=0.001), as well as three rows (F(2,93)=5.69, p=0.005). The Tukey post hoc analysis showed that the columns and rows could respectively be categorized into two distinct temperature regions (Table 3). For the longitudinal columns, there was no significant difference between the anteromedial and anterolateral columns. Likewise, there was no significant difference between the posteromedial and posterolateral columns. However, there was a significant difference between the anterior and posterior columns. For the circumferential rows, the distal row had a significant difference from the proximal and middle rows. However, there was no significant difference between the middle and proximal rows.

**Table 3:** The recorded temperature (Mean±SD) at each region over the residual limb

Columns	Anteromedial	Anterolateral	Posteromedial	Posterolateral	*F(3,92)*	P-value
28.4(SD=1.5)	28.4(SD=0.5)	26.9(SD=0.1)	27.2(SD=0.5)	6.1	0.001
Rows	Proximal		Middle	Distal	*F(2,93)*	P-value
	28.3(SD=1.6)		28.0(SD=1.7)	26.9(SD=1.7)	5.7	0.005
Classification of temperature sites based on their column and row (Note: sites under each class have no significant difference with each other. However, there is a significant difference (*p<0.05*) between classes)						
Temperature in Columns				Temperature in Rows		
Class 1		Class 2		Class 1		Class 2
Anteromedial		Posteromedial		Zone 1: Proximal		Zone 3: Distal
Anterolateral		Posterolateral		Zone 2: Middle		

### Relationship of average residual limb temperature with clinical and demographic characteristics of participants

Table 4 presents the correlation data between average residual limb skin temperature with participant demographic and clinical characteristics. There was no significant relationship between average residual limb temperature and participants’ demographic or clinical characteristics.

**Table 4:** Correlation of average residual limb temperature with clinical & demographic characteristics of participants.

	Statistics Value	Clinical & Demographic Characteristics								
		Quantitative^¥^						Nominal^‡^		
		Age (y)	Mass (kg)	Height (cm)	Time after Amputation (y)	Years of Prosthesis Use (y)	Daily Prosthesis Use (h)	Prosthesis Type	Employment Status	Amputation Side
Residual Limb Temperature	Correlation	0.45	0.33	-0.07	0.56	0.55	0.30	0.15	0.28	0.16
	p	0.27	0.42	0.88	0.15	0.16	0.48	0.66	0.18	0.32

¥: Pearson's r

‡: Partial Eta Square

## DISCUSSION

The present study focused on the temperature measurement of the TRL while outside of the prosthetic socket. On average, lower residual limb temperature was found compared to a previous similar study.^20^ Uneven temperature distribution over the TRL was found, which followed a specific thermal gradient pattern. The highest and lowest skin temperatures were recorded at the middle and distal zones of the anteromedial region of TRL, respectively. Skin temperature recording showed that the anterior part of the residual limb had significantly higher skin temperature compared to its posterior part. Similarly, the distal part of the residual limb had a significantly lower temperature than its middle and proximal parts.

### Temperature measurement over the transtibial residual limb

Uneven temperature distribution over TRL was found. The highest temperature (29.8 (SD=0.9)°C) was recorded in the middle part of the anteromedial (Z2) of TRL. The lowest temperature (26.3 (SD=0.9)°C) was recorded at the distal part of the anteromedial (Z3) of TRL.

Transtibial amputation is associated with the shortened length of the lower limb, which directly affects the amount of heat transfer by conduction and radiation. Likewise, the less surface area of the lower limb leads to reduced temperature transfer by evaporation. Furthermore, compromised muscles and blood vessels have lower potential in temperature transfer by convection. Therefore, different thermal patterns over the TRL could be anticipated compared to the sound limb. For instance, the highest and lowest temperature sites over the TRL are closer to each other than those shown by Gatt et al. in sound limbs using a thermography camera.^26^ The distance between the highest and lowest skin temperature would be highlighted whenever the temperature difference passes a physiological safe limit, leads to thermal discomfort, and jeopardizes skin integrity.^5,27^

Comparison of temperature recording of the TRL in the current study with previous studies^5,8,17,20^ is difficult considering the difference in thermography protocol. As earlier indicated, we recorded temperature while the residual limb was outside of a prosthetic socket. However, in previous studies, the temperature recording of the TRL was performed when the residual limb was inside the prosthetic socket. Our findings are comparable to the baseline phase of the thermography protocol in the Ghoseiri et al. study. They measured residual limb skin temperature in a single transtibial amputee. They found that the middle part of the anterolateral region of the TRL showed the highest skin temperature, whereas the distal part of the posterior region of the residual limb showed the lowest skin temperature.^20^ In line with our results, they showed uneven temperature distribution over the TRL skin, revealed warmer anterior columns than posterior ones, and spotted the colder region of the TRL at its distal end than the proximal end. However, the location of the warmest and coldest sites differs from the current study. This disagreement could be attributed to the difference in study designs and the number of recording sensors. Ghoseiri et al. recorded temperature in a reversal single-subject design with six thermistors over the TRL skin.^20^ The highest and lowest temperature sites of the TRL in those studies recorded temperature inside the socket were different from our findings. Peery et al. reported that the proximal anterior region of the residual limb was the coldest site and the posterior region of the residual limb was the warmest site.^8^ Klute et al. found that the middle part of the anterolateral location of the residual limb had the warmest temperature, while the posteromedial part of the distal end of the TRL had the coldest temperature.^5^

In the current study, the whole TRL’s average temperature was 27.7 (SD=0.8)°C, ranging from 27 to 31°C. This temperature was lower than the average temperature reported by Ghoseiri et al., 29.1 (SD=0.6)°C,^20^ probably because of the difference in study design and the number of thermistors. Although difficult to do this comparison, the average temperature of the whole TRL in current study was lower than amounts reported for inside socket thermography protocols, 29.5 (SD=0.9)°C,^17^ 31.0 (SD=1.5)°C,^5^ and 31.4 (SD=1.3)°C,^8^ probably because of the insulating characteristics of the prosthetic socket and liner that contributed to higher residual limb temperature, as well as temperature measuring following periods of activity.

It was reported that localized higher skin temperature than the adjacent parts is a predictor of skin damage.^27^ Therefore, maintenance or provision of a constant temperature, thermoregulation, by keeping a relatively constant temperature and heating or cooling mechanisms could ensure optimal physiological health and function.^28^ Thermoregulation can be induced internally (e.g., by changes in the blood flow during vasodilation or vasoconstriction)^29^ or externally (e.g., thermoregulatory systems and exercise maneuvers).^20^ With respect to the external thermoregulation, the pattern of temperature distribution over the TRL may be useful for selecting appropriate thermoregulatory strategies both in and out of the prosthesis. Challenges in developing a thermoregulatory system include managing the size, weight, cost, and required power to efficiently work when applied as a prosthetic component.^5,20^ Therefore, for both in and out of prosthesis approaches, the distinct skin temperature measurements based on column and row could help to select the best attachment sites of thermoregulatory systems. Our findings revealed that the anteromedial and posteromedial columns of the residual limb were the warmest and coldest regions, respectively, while the anterior part of the TRL had higher temperature compared to the posterior part. Furthermore, the proximal and middle circumferential rows had higher temperature compared to the distal row. Therefore, to provide a thermal equilibrium, i.e., balance between the rate of heat production and rate of heat release, out of the prosthesis, a cooling mechanism may be required for the proximal and middle rows of the anterior part of the residual limb, while a heating mechanism may be necessary for the distal and posterior parts of the residual limb. For instance, therapeutic exercises which showed promising cooling effects in able-bodied people could be modified and used in people with amputation. However, this concept needs further investigation.

Thermal standards in able-bodied people are based on both environmental (e.g., air temperature, air velocity, radiant temperature, and relative humidity) and personal factors (e.g., activity level, metabolic rate, the weighted average of skin temperature, and clothing insulation).^29,30^ In people with amputation, the residual limb skin temperature is generally greater than in able-bodied people because of the decreased surface area of the body and changes in blood circulation and the volume and shape of the residual limb muscles.^21,31^ Interestingly, the distal end of the residual limb in unilateral amputees is cooler than the corresponding site on the contralateral sound side, probably due to less blood flow, damaged vessels, fat accumulation, and more skin surface due to amputation consequences.^9,21^ Therefore, because of many different factors between able-bodied and amputees, thermal standards available for able-bodied people cannot be used for people with amputation.

### Relationship of the average residual limb temperature with demographic and clinical characteristics of participants

Statistical analysis revealed small non-significant relationships between the whole residual limb temperature and participants’ demographic and clinical characteristics. This finding could highlight the importance of the socket barrier and physical activity in increasing the residual limb skin temperature.^3^ Indeed, our thermography protocol of temperature recording at the rest condition and outside of a prosthetic socket leads to opposite findings against the general belief that age, lifestyle, and physical condition by impacting the metabolism and perfusion rate of the blood alters temperature distribution pattern of the TRL^9^ and thermal discomfort.^18^ We found that the middle part of the anteromedial region of the residual limb had an over 2°C temperature difference with the residual limb’s mean temperature; therefore, this site may have a higher vulnerability to thermal discomfort and skin irritation. With increasing age, the thermal sense may be decreased, and some older persons may not detect up to 4°C of temperature change.^32^ However, thermal sense quantification differs from skin temperature recording and is beyond the scope of the present study. Although the present study had no focus on participants’ thermal comfort, Klute et al. reported an increase of 2°C could cause thermal discomfort in people with amputation.^5^ In contrast, Diment and colleagues noted that thermal discomfort in lower limb prosthetic users is not directly related to the skin temperature.^33^ Similar to localized TRL skin temperature, there is no consensus about thermal comfort in people with amputation.

The interaction of residual limb skin temperature with demographic and clinical characteristics could affect the quality of life in people with amputation. Thus, it needs further investigation, probably around residual limb tissue characteristics.

### Study Limitations

Several aspects may threaten the internal and external validity of this study. Evaluating residual limb temperature is difficult because the thermistors are connected to a computer or microcontroller using small and breakable wires, likely leading to small sample sizes in the previous temperature measurement studies in amputees.^5,8,17,20^ For instance, the sample size in Peery et al., Klute et al., Huff et al., and Ghoseiri et al. were 5, 9, 1, and 1, respectively.^5,8,17,20^ The small sample size and purposive sampling of male adult traumatic (war-related) amputees may limit the generalizability of the results to females and those who suffered amputation following dysvascularity and other etiologies. Future studies may assess thermoregulatory mechanisms such as skin perfusion or thermal receptor activation. Skin and muscle thickness were not evaluated in this study, which may influence results in two different ways. High skin thickness could decrease heat transfer by conduction, which potentially leads to reduced surface temperature. In contrast, the increased muscle thickness could imply more metabolic rate, higher temperature production, and higher power for pumping blood in the vascular system, all associated with higher skin temperature. Therefore, future research may use musculoskeletal ultrasound to quantify the soft tissue thickness of the residual limb. Although it is beyond the scope of the present study, from a surgical standpoint, there are two primary techniques for transtibial amputation, i.e., myodesis and myoplasty, which respectively being indicated for traumatic and dysvacular amputees. In myodesis, muscle attaches to the bone, while in myoplasty, muscle sutures to another muscle. Therefore, there would be some difference in the length-tension relationship of the muscles and blood flow rate at the residual limb.^34^ Thus, an investigation of the chosen amputation technique on TRL temperature distribution is warranted. A direct comparison between residual and sound limbs on the same participant would expand the baseline knowledge of temperature distribution in TRL.

## CONCLUSION

This study may provide important information to develop thermoregulatory strategies for the residual limb in transtibial amputees, ranging from prosthetic socket design, component manufacturing, and material selection to potential therapeutic exercises. Thermoregulatory strategies need to address the unequal skin temperature distribution over the TRL while outside the prosthetic socket. Provision thermal equilibrium needs cooling and heating mechanisms for anterior and posterior regions of the TRL, respectively. Likewise, a heating mechanism for the distal part, and cooling mechanisms for the middle and proximal parts of the TRL. Temperature recording revealed that the highest and lowest skin temperatures were located at the middle and distal zones of the anteromedial region of TRL, respectively. Thus, localized thermoregulatory strategies could address heating/cooling mechanisms to prevent thermal-related skin irritation. Further thermoregulatory investigations (both in and out of the socket) with larger sample sizes and inclusion of different groups of people with transtibial amputation are warranted; these studies should consider the volume and thickness of the skin and muscles, as well as the blood perfusion rate at different regions of the residual limb.

## DECLARATION OF CONFLICTING INTERESTS

The authors declare that they have no competing interests,

## AUTHOR CONTRIBUTION

**Kamiar Ghoseiri:** contributed to the study concept and design, participated in data gathering, analyzed and interpreted data, contributed to the drafting of the manuscript and read and approved the final manuscript.

**Mostafa Allami:** contributed to the study concept and design, participated in data gathering, contributed to the drafting of the manuscript and read and approved the final manuscript.

**Justin R. Murphy:** analyzed and interpreted data, contributed to the drafting of the manuscript and read and approved the final manuscript.

**Phillip Page:** analyzed and interpreted data, contributed to the drafting of the manuscript and read and approved the final manuscript.

**Duane C. Button:** contributed to the study concept and design, analyzed and interpreted data, contributed to the drafting of the manuscript and read and approved the final manuscript.

## SOURCES OF SUPPORT

This material was based on the work supported by the Vice-chancellor for Research and Technology, Hamadan University of Medical Sciences (No. 950304954).

## ETHICAL APPROVAL

All aspects of the study were approved by the ethics committee of the Hamadan University of Medical Sciences (IR.UMSHA.REC. 1394.333). After a full description of the study aims and procedures, written informed consent was obtained from participants before enrollment.
